# Magnesium deficiency prevents high-fat-diet-induced obesity in mice

**DOI:** 10.1007/s00125-018-4680-5

**Published:** 2018-07-09

**Authors:** Steef Kurstjens, Janna A. van Diepen, Caro Overmars-Bos, Wynand Alkema, René J. M. Bindels, Frances M. Ashcroft, Cees J. J. Tack, Joost G. J. Hoenderop, Jeroen H. F. de Baaij

**Affiliations:** 1 Department of Physiology (286), Radboud Institute for Molecular Life Sciences, Radboud university medical center, P. O. Box 9101, 6500 HB Nijmegen, the Netherlands; 2 Department of Internal Medicine, Radboud Institute for Molecular Life Sciences, Radboud university medical center, Nijmegen, 6500 HB the Netherlands; 3 Centre for Molecular and Biomolecular Informatics, Radboud Institute for Molecular Life Sciences, Radboud university medical center, Nijmegen, 6500 HB the Netherlands; 4 Department of Physiology, Anatomy & Genetics, University of Oxford, Oxford, UK

**Keywords:** β-Adrenergic receptor, Brown adipose tissue, Energy homeostasis, Hypomagnesaemia, Lipid metabolism, Lipolysis, Magnesium, Obesity, White adipose tissue

## Abstract

**Aims/hypothesis:**

Hypomagnesaemia (blood Mg^2+^ <0.7 mmol/l) is a common phenomenon in individuals with type 2 diabetes. However, it remains unknown how a low blood Mg^2+^ concentration affects lipid and energy metabolism. Therefore, the importance of Mg^2+^ in obesity and type 2 diabetes has been largely neglected to date. This study aims to determine the effects of hypomagnesaemia on energy homeostasis and lipid metabolism.

**Methods:**

Mice (*n* = 12/group) were fed either a low-fat diet (LFD) or a high-fat diet (HFD) (10% or 60% of total energy) in combination with a normal- or low-Mg^2+^ content (0.21% or 0.03% wt/wt) for 17 weeks. Metabolic cages were used to investigate food intake, energy expenditure and respiration. Blood and tissues were taken to study metabolic parameters and mRNA expression profiles, respectively.

**Results:**

We show that low dietary Mg^2+^ intake ameliorates HFD-induced obesity in mice (47.00 ± 1.53 g vs 38.62 ± 1.51 g in mice given a normal Mg^2+^-HFD and low Mg^2+^-HFD, respectively, *p* < 0.05). Consequently, fasting serum glucose levels decreased and insulin sensitivity improved in low Mg^2+^-HFD-fed mice. Moreover, HFD-induced liver steatosis was absent in the low Mg^2+^ group. In hypomagnesaemic HFD-fed mice, mRNA expression of key lipolysis genes was increased in epididymal white adipose tissue (eWAT), corresponding to reduced lipid storage and high blood lipid levels. Low Mg^2+^-HFD-fed mice had increased brown adipose tissue (BAT) *Ucp*1 mRNA expression and a higher body temperature. No difference was observed in energy expenditure between the two HFD groups.

**Conclusions/interpretation:**

Mg^2+^-deficiency abrogates HFD-induced obesity in mice through enhanced eWAT lipolysis and BAT activity.

**Electronic supplementary material:**

The online version of this article (10.1007/s00125-018-4680-5) contains peer-reviewed but unedited supplementary material, which is available to authorised users.

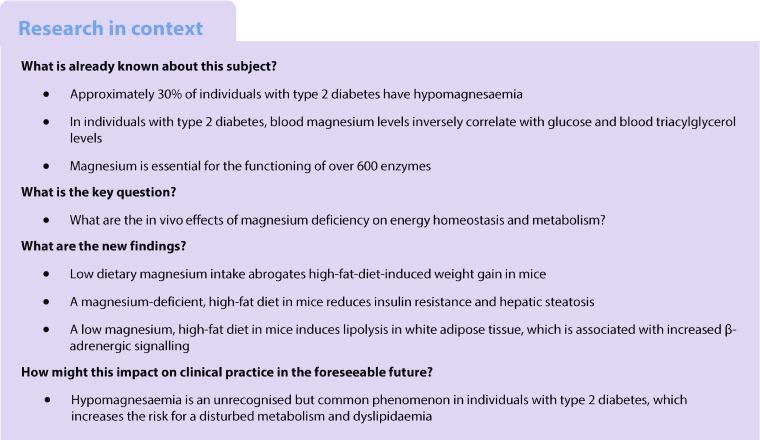



## Introduction

Hypomagnesaemia (blood Mg^2+^ concentration <0.7 mmol/l) affects approximately 30% of individuals with type 2 diabetes [1, 2]. Hypomagnesaemia is an important risk factor for the development and progression of type 2 diabetes [3–5]. Low dietary Mg^2+^ intake and reduced serum Mg^2+^ concentrations have also been associated with obesity, although with conflicting results [1, 6–8]. Moreover, reduced blood Mg^2+^ levels have been correlated with elevated glucose and triacylglycerol concentrations in individuals with type 2 diabetes, suggesting that hypomagnesaemia is associated with insulin resistance and dyslipidaemia [1].

Mg^2+^ fulfils many roles including cell growth, membrane stability, enzyme activity and energy metabolism [9]. It is a cofactor for numerous enzymes, primarily because it stabilises ATP and facilitates phosphate transfer reactions [10, 11]. Mg^2+^ is essential for glycolysis and the citric acid cycle [12, 13]. Because Mg^2+^ is critical for insulin receptor tyrosine kinase activity, hypomagnesaemia has also been implicated in insulin resistance [14–16]. Recently, hypomagnesaemia in mice was shown to contribute to enhanced catabolism, but no in-depth metabolic phenotype analysis was performed [17].

In type 2 diabetes, restoring serum Mg^2+^ values by oral Mg^2+^ supplementation improves insulin sensitivity, decreases fasting glucose levels [18] and corrects the lipid profile [19–21]. Although Mg^2+^ is essential for key enzymes in lipid metabolism, including hepatic lipase and lecithin-cholesterol acyltransferase [22, 23], the effects of chronic Mg^2+^ deficiency on adipocyte function and lipid metabolism remain largely unknown.

In this study, we explored the role of Mg^2+^ in energy homeostasis, insulin sensitivity and lipid metabolism, by feeding mice a low-fat diet (LFD) or a high-fat diet (HFD) combined with low or normal Mg^2+^ for 17 weeks. The resulting metabolic effects were extensively characterised. Data were confirmed by an independent replication experiment.

## Methods

### Seventeen-week mouse study: Radboud university medical center

This study was approved by the animal ethics board of the Radboud University Nijmegen (RU DEC 2015-0073) and the Dutch Central Commission for Animal Experiments (CCD, AVD103002015239). Forty-eight male C57BL6/J mice (Charles River Laboratories, Sulzfeld, Germany), aged 9–10 weeks, were randomly allocated to four experimental groups of *n* = 12 mice. Experimental diets consisted of 10% or 60% energy from palm oil plus 0.03% or 0.21% wt/wt magnesium oxide. Researchers and animal caretakers were blinded for Mg^2+^ content. On days −1, 84 and 112, mice were housed individually in metabolic cages for 24 h. Blood was collected via cheek puncture at days −1, 28, 56 and 84. At weeks 14 and 15, ITT and GTT, respectively, were performed. After 17 weeks on the diets, mice were anaesthetised by 4% vol./vol. isoflurane and exsanguinated via orbital sinus bleeding. See electronic supplementary material (ESM) for full methods.

### Intraperitoneal insulin and glucose tolerance tests

After 14 weeks on the diets, mice underwent an intraperitoneal ITT. After 6 h of fasting, from 08:00 to 14:00, mice were injected with 0.75 U/kg body weight of human insulin (Novorapid, Novo Nordisk, Bagsværd, Denmark). Blood glucose levels were measured at 0, 20, 40, 60, 90 and 120 min. After 15 weeks on the diets, mice underwent an IPGTT. After an overnight fast, from 18:00 to 09:00, mice were injected with 2 g/kg body weight of d-glucose (Invitrogen, Bleiswijk, the Netherlands). Blood glucose was measured at 0, 15, 30, 60 and 120 min. See ESM for full methods.

### Quantitative real-time PCR

Total RNA was extracted using TRIzol reagent (Invitrogen, Paisley, UK), subjected to DNase (Promega, Fitchburg, WI, USA) treatment and measured using the Nanodrop 2000c spectrophotometer (Thermo Scientific, Waltham, MA, USA). RNA was reverse transcribed using Moloney murine leukaemia virus (M-MLV) reverse transcriptase (Invitrogen, Bleiswijk, the Netherlands). Gene expression levels were quantified by SYBR-Green (Bio-Rad, Veenendaal, the Netherlands) on a CFX96 real-time PCR detection system (Bio-Rad) and normalised for *Gapdh*. Primer sequences for *Acadl*, *Adrb3*, *Atgl* (also known as *Pnpla2*), *Cact*, *Cd36*, *Cpt1-l* (also known as *Cpt1a*), *Cpt1-m* (also known as *Cpt1b*), *Cpt2*, *Fbp1*, *G6pase* (also known as *G6pc*), *Gapdh*, *Glut1* (*also known as Slc2a1*), *Glut2* (also known as *Slc2a2*), *Glut4* (also known as *Slc2a4*), *Gs*, *Gyk*, *Hmgcs1*, *Hsl* (also known as *Lipe*), *Mgll*, *Pepck1*, *Pklr*, *Ppar-α* (also known as *Ppara*), *Ppar-γ* (also known as *Pparg*), *Srebp1* (also known as *Srebf1*) and *Ucp1* are provided in ESM Table 1.

### Histology

Epididymal fat and liver tissues were fixed in 10% vol./vol. neutral-buffered formalin (KLINIPATH, Duiven, the Netherlands) in PBS. Samples were dehydrated through alcohol, embedded in paraffin and cut into 4 μm sections. Sections were stained with H&E using standard procedures. The average cell size of 100–300 cells per mouse was determined manually using ImageJ software (v1.48, NIH, Bethesda, MD, USA, RRID:SCR_003070). Liver samples were snap frozen in liquid nitrogen, cut into 10 μm sections, stained with Oil Red O (Sigma-Aldrich, St. Louis, MO, USA) and counterstained with haematoxylin.

### RNA sequencing

Five randomly selected samples of each group were analysed, with no technical replicates, by RNA sequencing. Quality control and RNA sequencing were performed by the Beijing Genomics Institute (BGI), Hong Kong, China. Per sample, 13 million reads were sequenced using the Hiseq 4000 platform (Illumina, San Diego, CA, USA) using a 50 bp single-end module. Clean reads were mapped to *Mus musculus* transcriptome (GRCm38/mm10) using the HISAT/Bowtie2 tool (RRID:SCR_005476) [24, 25]. RSEM software v1.2.31 (RRID:SCR_013027) was used to quantify gene expression levels (fragments per kilobase million [FPKM] values) [26]. FPKM values were log_2_ transformed and further analysed in R (www.r-project.org, v3.4.1., RRID:SCR_001905). Heatmaps for individual GO terms were created using the ggplot2 library (r-project) [27]. See ESM for full method details.

### Analytical procedures

Serum Mg^2+^ was determined using a spectrophotometric assay at 600 nm (Roche/Hitachi, Tokyo, Japan) according to the manufacturer’s protocol. Liver samples were weighed and lysed in lysis buffer (10% wt/vol.) containing 50 mmol/l Tris-HCl pH 7.5, 1 mmol/l EGTA, 1 mmol/l EDTA, 1% vol./vol. Triton X-100, 10 mmol/l glycerophosphate, 1 mmol/l sodium orthovanadate, 50 mmol/l sodium fluoride, 10 mmol/l sodium pyrophosphate and 150 mmol/l sodium chloride. Triacylglycerol concentrations in serum and liver lysate were assayed using an enzymatic kit (Roche Molecular Biochemicals, Indianapolis, IN, USA), according to the manufacturer’s protocol. Serum NEFA (NEFA-C kit, WAKO Diagnostics, Delfzijl, the Netherlands), cholesterol (Human Diagnostics, Wiesbaden, Germany), glucose (Instruchemie, Delfzijl, the Netherlands), leptin (R&D Systems, Minneapolis, MN, USA) and adiponectin (R&D Systems, Minneapolis, MN, USA) concentrations were determined according to manufacturers’ protocols.

3-Methoxytyramine and normetanephrine were analysed by a 6490 LC-MS/MS (Agilent Technologies, Amstelveen, the Netherlands) after solid phase extraction (SPE) Oasis WCX μElution sample cleanup (Waters, Etten-Leur, the Netherlands). A calibration curve was used with 3-methoxytyramine-HCl and normetanephrine-HCl (Sigma-Aldrich, St. Louis, MO, USA) as calibrators. 3-Methoxytyramine-d_4_-HCl and normetanephrine-d_3_-HCl (Medical Isotopes, Pelham, NH, USA) were used as internal standards. An ethylene bridged hybrid (BEH) Amide 1.7 μm 100A, 2.1 × 100 mm column (Waters) was used as an analytical column.

### Nine-week replication mouse study: MRC Harwell Institute

All experimental procedures were conducted in compliance with the UK Animals Scientific Procedures Act (1986) and University of Oxford ethical guidelines. Thirty-nine male C57BL6/J mice (Medical Research Council [MRC], Harwell, UK) were randomly allocated to four groups of *n* = 10 mice (*n* = 9 in the low Mg^2+^[LowMg]-LFD group). At 8 weeks old, mice were put on experimental diets identical to the Radboud university medical center experiment for 9 weeks. At day 14, mice were housed individually in metabolic cages (Tecniplast, Buguggiate, Italy). Blood was collected via tail bleed at days −1 and 14. Respiration metabolic cages (TSE PhenoMaster Cages, Bad Homburg, Germany) were used at days 28 and 56 and body temperatures were measured by rectal probe (ATP-instrumentation, Ashby, UK). Data were averaged per hour and plotted from 18:30 to 09:30 h. See ESM Methods for full details.

### Lipolysis in 3 T3-L1 adipocytes

Differentiated 3 T3-L1 cells (mycoplasma-free, ATCC, Manassas, VA, USA) were incubated for 20 h in DMEM, containing 0 or 1 mmol/l MgCl_2_. Aliquots of 50 μl medium were taken hourly and heated for 8 min at 65°C. The concentration of NEFA was assessed using the WAKO NEFA-C kit (Instruchemie, Delfzijl, the Netherlands). See ESM Methods for full details.

### Statistics

For the animal experiments, a two-way ANOVA was used to look for a significant interaction effect between the two main variables (dietary fat and Mg^2+^ content). If there was none, significant differences between the groups were assessed using a two-way ANOVA approach with a Tukey’s multiple comparisons test. If the two-way ANOVA demonstrated a significant interaction effect between the two main variables, an unpaired multiple *t* test approach using the Holm–Sidak method for multiple comparisons was used. Statistical significance was determined using Graphpad Prism v7 (La Jolla, CA, USA, RRID: SCR_002798). For the lipolysis assays, an unpaired Student’s *t* test was used.

Differences with a *p* value of <0.05 were considered statistically significant. Results are presented as mean ± SEM.

## Results

### Low dietary Mg^2+^ intake reduces diet-induced obesity in mice

**Fig. 1:**
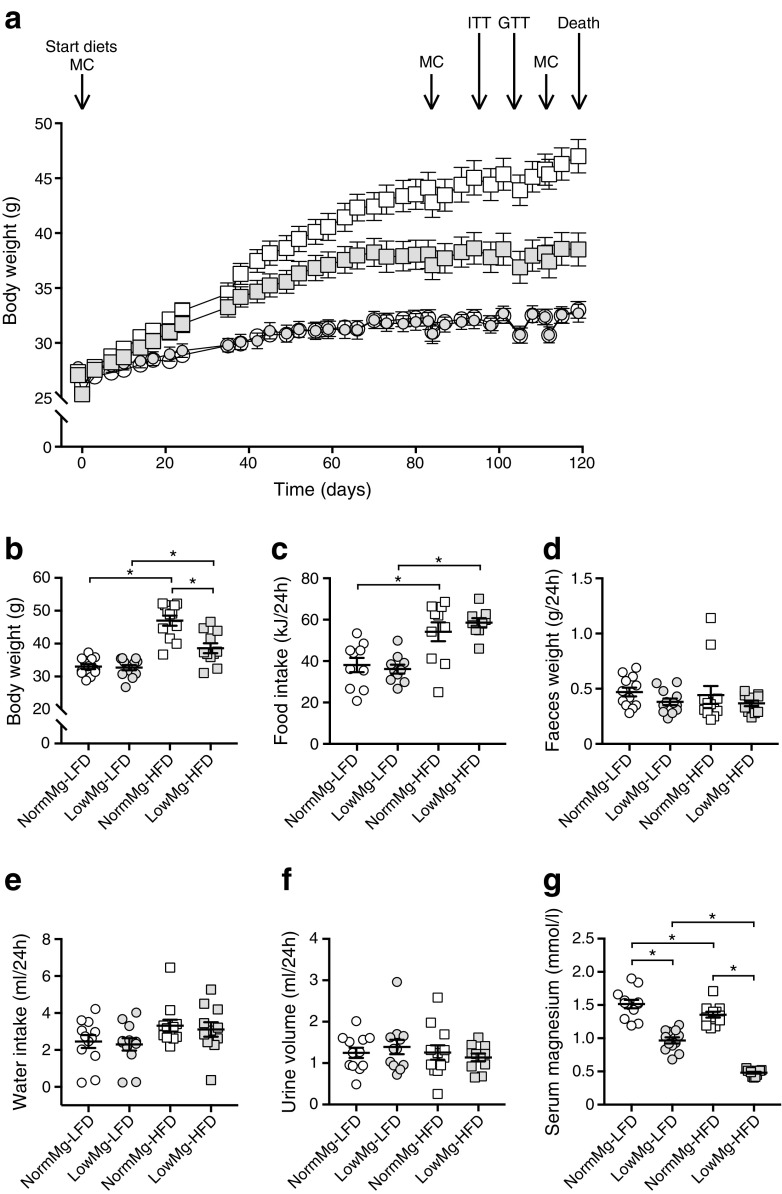
Low dietary Mg^2+^ intake reduces diet-induced obesity in mice. Adult mice (*n* = 12 mice per group, *n* = 11 mice for the LowMg-HFD group) were fed a diet with either an LFD or an HFD, combined with a normal or low Mg^2+^ concentration, for 17 weeks. (**a**) Body weight, determined twice weekly. Arrows indicate experimental interventions: metabolic cage (MC), ITT and GTT. (**b**) The body weight of mice at week 17 (the end of the experiment). (**c**) Food intake, (**d**) total faeces weight, (**e**) water intake (two-way ANOVA for dietary fat effect *p* < 0.05) and (**f**) urine production determined over a period of 24 h, using metabolic cages, at week 16. (**g**) Serum Mg^2+^ levels at death. NormMg-LFD (white circles), LowMg-LFD (grey circles), NormMg-HFD (white squares), LowMg-HFD (grey squares). Data are mean ± SEM. Depending on the absence or presence of a significant interaction effect between dietary fat and Mg^2+^ content, either a two-way ANOVA (Tukey’s multiple comparison test) or an unpaired multiple *t* test (Holm–Sidak multiple comparison test) approach, respectively, was used to determine statistical significance. *p < 0.05 for the comparisons shown

The mice were fed an LFD or HFD containing either a low (0.03% wt/wt) or normal (0.21% wt/wt) Mg^2+^ concentration for 17 weeks (Fig. 1a). There was no difference in body weight between low and normal Mg^2+^ groups on the LFD, but mice on the LowMg-HFD gained significantly less weight than those on the normal Mg^2+^(NormMg)-HFD (47.00 ± 1.53 g vs 38.62 ± 1.51 g in mice given a NormMg-HFD and LowMg-HFD, respectively, *p* < 0.05, Fig. 1a,b). The lower body weight of the LowMg-HFD group could not be explained by differences in dietary intake, as shown by similar food intake and faeces production between the two HFD groups (Fig. 1c,d). There was also no difference in water intake or urinary volume between the HFD groups (Fig. 1e,f). Hypomagnesaemia was detected in both the LowMg groups, but was significantly more pronounced in mice that were concomitantly fed an HFD (Fig. 1g).

### Reduced diet-induced obesity in Mg^2+^-deficient mice is accompanied by improved insulin sensitivity

**Fig. 2:**
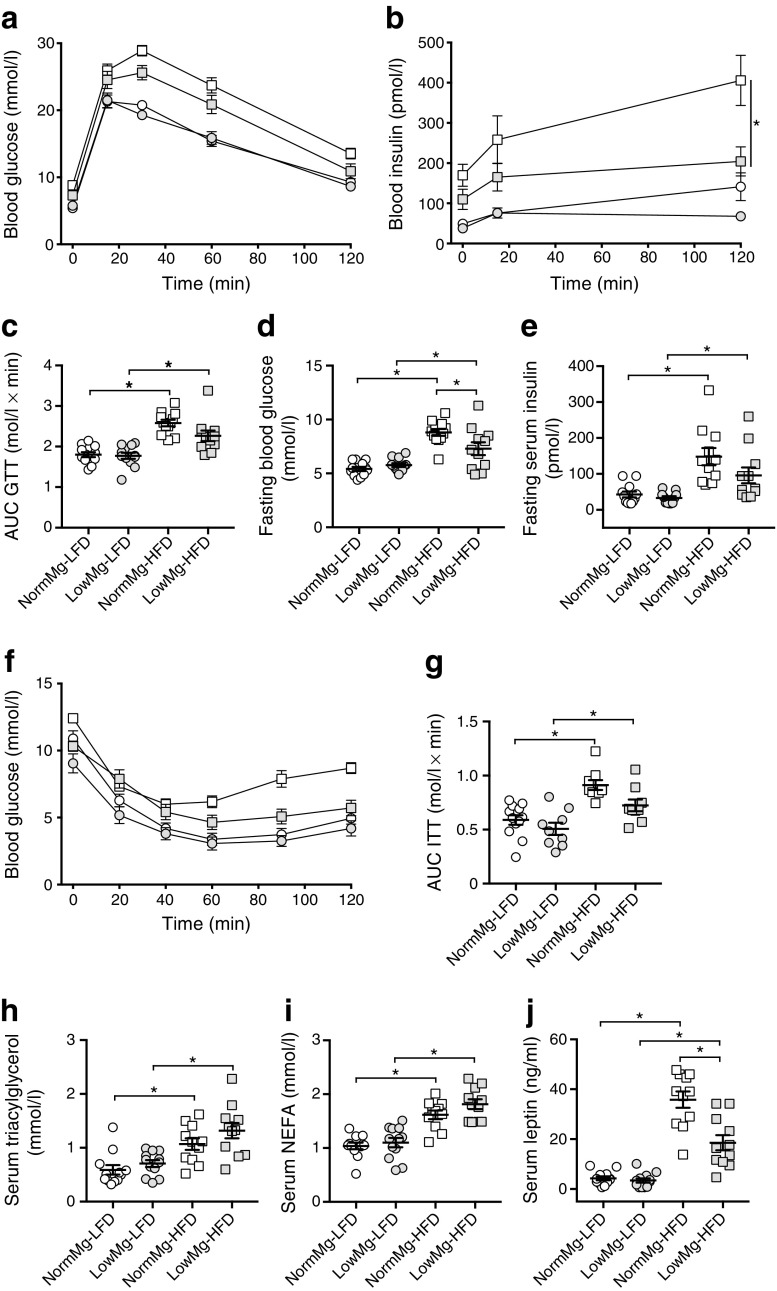
Reduced diet-induced obesity in Mg^2+^-deficient mice is accompanied by high triacylglycerol levels and improved insulin sensitivity. (**a**) Glucose clearance determined by IPGTT at week 15. (**b**) Serum insulin measured at 0, 15 and 120 mins of the GTT. (**c**) AUC determined between 0 and 120 min. (**d**) Fasting blood glucose and (**e**) serum insulin measured during the GTT. (**f**) Blood glucose measured during an IPITT at week 14 (*n* = 9 mice per group, *n* = 12 for the NormMg-LFD. (**g**) AUC determined between 0 and 120 min (two-way ANOVA for dietary Mg^2+^ effect, *p* < 0.05). Non-fasted serum (**h**) triacylglycerol, (**i**) NEFA and (**j**) leptin concentrations at death (*n* = 12 mice for both LFD groups, *n* = 11 mice for both HFD groups). NormMg-LFD (white circles), LowMg-LFD (grey circles), NormMg-HFD (white squares), LowMg-HFD (grey squares). Data are mean ± SEM. Depending on the absence or presence of a significant interaction effect between dietary fat and Mg^2+^ content, either a two-way ANOVA (Tukey’s multiple comparison test) or an unpaired multiple *t* test (Holm–Sidak multiple comparison test) approach, respectively, was used to determine statistical significance. *p < 0.05 for the comparisons shown

To explore glucose metabolism in more detail, beta cell function and insulin sensitivity were determined by IPGTT and IPITT. In the IPGTT (a measure for beta cell dysfunction and insulin resistance), glucose clearance was reduced in both HFD groups (Fig. 2a). Glucose clearance was not significantly different between NormMg-HFD-fed mice and LowMg-HFD-fed mice (2.58 ± 0.08 vs 2.26 ± 0.13 mol/l × min in NormMg-HFD- and LowMg-HFD-fed mice, respectively, *p =* 0.07, Fig. 2c). LowMg-HFD-fed mice required significantly less insulin than NormMg-HFD-fed mice to clear the glucose, consistent with LowMg-HFD-fed mice being more insulin sensitive (Fig. 2b). Fasting blood glucose and insulin concentrations were significantly increased in the HFD-fed mice, in accordance with the increased body weight (Fig. 2d,e). Compared with the NormMg-HFD-fed mice, fasting blood glucose was lower in the LowMg-HFD-fed mice (Fig. 2d). The effect of dietary Mg^2+^ on fasting insulin was not statistically significant (Fig. 2e, two-way ANOVA for dietary Mg^2+^ effect, *p* = 0.07).

Both HFD-fed groups demonstrated increased insulin resistance in the ITT compared with their respective controls (Fig. 2f,g). Low dietary Mg^2+^ content resulted in a significantly lower AUC of the ITT (Fig. 2g, two-way ANOVA for dietary Mg^2+^ effect *p* < 0.05). In the LowMg-HFD group compared with the NormMg-HFD-fed mice, the AUC of the ITT was not significantly different (0.91 ± 0.05 vs 0.72 ± 0.05 mol/l × min in NormMg-HFD-fed and LowMg-HFD-fed mice, respectively, Tukey’s test *p* = 0.07, Fig. 2f,g).

Insulin resistance is often accompanied by hyperlipidaemia, in particular, high triacylglycerol and NEFA levels. As expected, the HFD-fed mice had higher serum triacylglycerol and NEFA levels than LFD-fed mice (Fig. 2h,i). Interestingly, despite their lower body weight and increased insulin sensitivity, LowMg-HFD-fed mice also had high serum triacylglycerol and NEFA (Fig. 2h,i). In contrast, serum leptin levels correlated with body weight; hence, reduced leptin levels were observed in the LowMg-HFD-fed mice compared with NormMg-HFD-fed mice (Fig. 2j). No difference between the two HFD groups was observed in serum adiponectin and cholesterol (ESM Fig. 1a,b).

### Mg^2+^ deficiency prevents diet-induced hepatic lipid storage

**Fig. 3:**
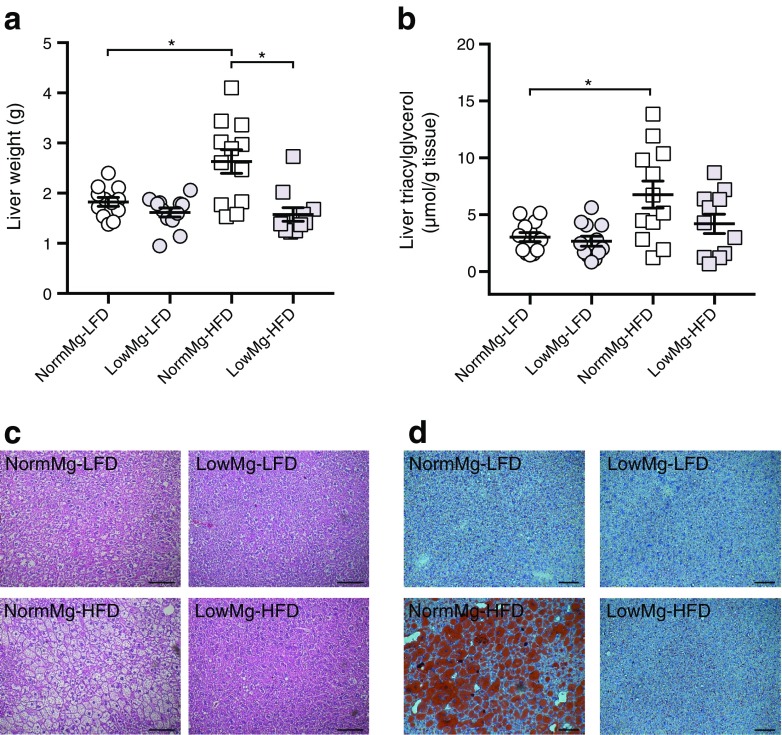
Mg^2+^ deficiency prevents diet-induced hepatic lipid storage. Liver (**a**) weight and (**b**) triacylglycerol content at death (17 weeks, *n* = 12 mice per group, *n* = 11 mice for the LowMg-HFD group). Representative images of livers stained with (**c**) H&E or (**d**) Oil Red O. Scale bars, 100 μm. NormMg-LFD (white circles), LowMg-LFD (grey circles), NormMg-HFD (white squares), LowMg-HFD (grey squares). Data are mean ± SEM. Depending on the absence or presence of a significant interaction effect between dietary fat and Mg^2+^ content, either a two-way ANOVA (Tukey’s multiple comparison test) or an unpaired multiple *t* test (Holm–Sidak multiple comparison test) approach was used to determine statistical significance, respectively. *p < 0.05 for the comparisons shown

Liver function is often impaired in type 2 diabetes as a consequence of insulin resistance and hepatic steatosis [28]. Feeding mice a NormMg-HFD resulted in a significantly heavier liver. However, this effect was abrogated in mice fed a LowMg-HFD (Fig. 3a). In Mg^2+^-deficient mice, the HFD did not increase liver triacylglycerol content (Fig. 3b). In line with the triacylglycerol measurements, H&E and Oil Red O staining showed reduced hepatic lipid accumulation in the Mg^2+^-deficient HFD-fed mice (Fig. 3c,d, respectively). Hepatic mRNA expression of *Cd36*, a long-chain fatty acid transporter, was reduced in the LowMg-HFD-fed mice compared with the NormMg-HFD-fed mice (ESM Fig. 2a). Low dietary Mg^2+^ increased hepatic mRNA expression of sterol regulatory element-binding protein 1c (*Srebp1c*), which is involved in cholesterol and fatty acid metabolism, and phosphoenolpyruvate carboxykinase 1 (*Pepck1*), essential for gluconeogenesis, glyceroneogenesis and fatty acid re-esterification (ESM Fig. 2b,c, two-way ANOVA for dietary Mg^2+^ effect *p* < 0.05). No differences were observed between the two HFD-fed groups in the expression of key genes involved in hepatic glycolysis, ketogenesis and β oxidation (ESM Fig. 2d–l).

### Reduced adipose tissue mass in Mg^2+^-deficient HFD-fed mice is associated with increased mRNA expression of lipolysis genes

**Fig. 4:**
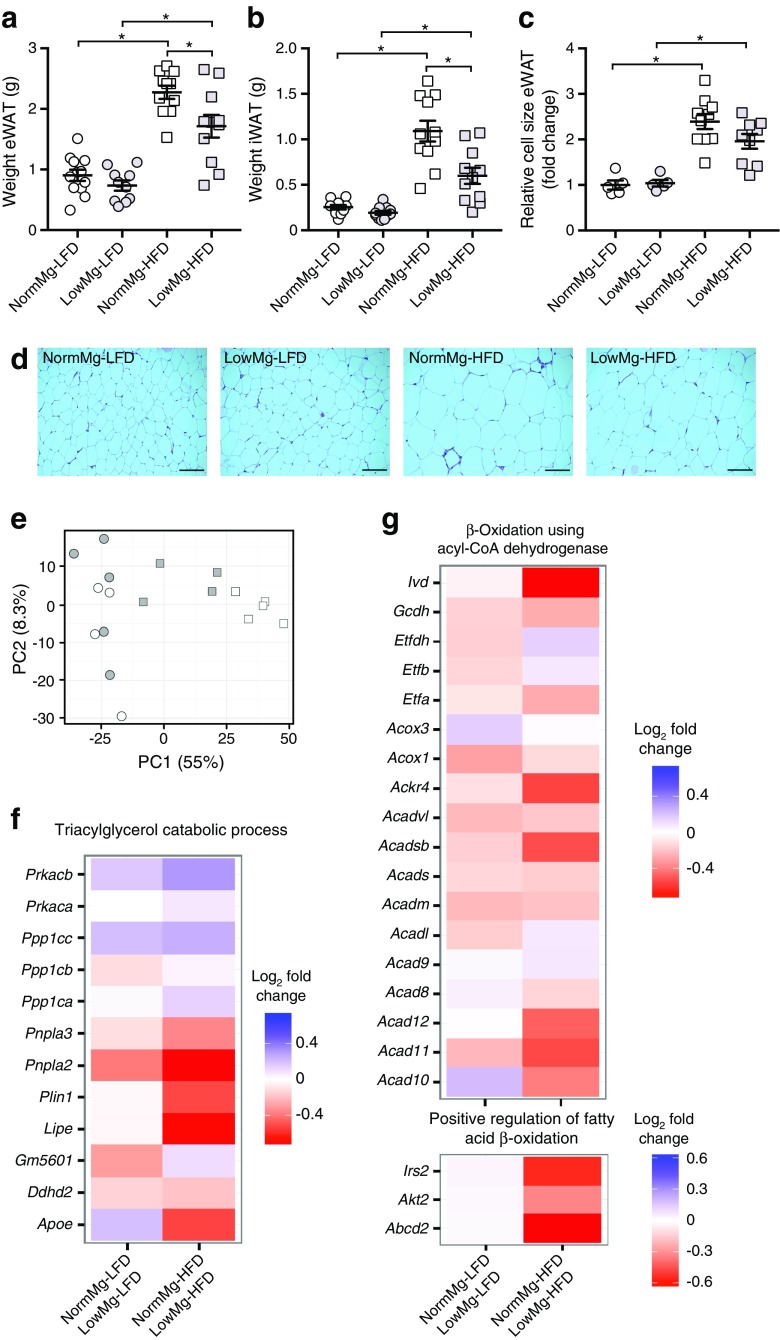
Reduced adipose tissue mass in Mg^2+^-deficient HFD-fed mice is associated with increased mRNA expression of lipolysis genes in eWAT. Weight of (**a**) eWAT, (**b**) iWAT and (**c**) eWAT cell size at death (17 weeks, *n* = 12 mice per group, *n* = 11 mice for the LowMg-HFD group). NormMg-LFD (white circles), LowMg-LFD (grey circles), NormMg-HFD (white squares), LowMg-HFD (grey squares). Data are mean ± SEM. Depending on the absence or presence of a significant interaction effect between dietary fat and Mg^2+^ content, either a two-way ANOVA (Tukey’s multiple comparison test) or an unpaired multiple *t* test (Holm–Sidak multiple comparison test) approach was used, respectively, to determine statistical significance. (**d**) Representative images of H&E stained eWAT. Scale bars, 100 μm. (**e**) Principal component (PC) analysis of RNA sequencing on eWAT. NormMg-LFD (white circles, *n* = 4), LowMg-LFD (grey circles, *n* = 5), NormMg-HFD (white squares, *n* = 5), LowMg-HFD (grey squares, *n* = 4). The percentages on the *x*-axis and *y*-axis indicate the total percentage of variance explained by the first two principal components, respectively. GO term analyses of the pathways (**f**) ‘triacylglycerol catabolic process’ and (**g**) ‘β-oxidation using acyl-CoA dehydrogenase’; and ‘positive regulation of fatty acid β-oxidation’. Gene expression changes are presented as log_2_ fold change with the NormMg^2+^ diet as reference, so that a negative value (in red) indicates a decrease in expression in the NormMg^2+^ vs LowMg^2+^ groups. *p < 0.05 for the comparisons shown

Our results show that mice fed a LowMg-HFD diet exhibit reduced body weight and high triacylglycerol levels compared with their NormMg-HFD-fed littermates. Interestingly, the LowMg-HFD group had decreased mass of epididymal and inguinal white adipose tissue (eWAT and iWAT, respectively) (Fig. 4a,b), which may point towards defective lipid handling in white adipose tissue (WAT). The HFD increased adipocyte size (Fig. 4c,d), but no significant difference was observed between the two HFD groups (Fig. 4c,d). Nevertheless, quantitative PCR showed that mRNA expression of *Srebp1c*, *Pepck1* and genes involved in β oxidation was increased in the eWAT of the LowMg-HFD group compared with the NormMg-HFD group (ESM Fig. 3a–f).

To determine the consequences of low Mg^2+^ on lipid metabolism, we performed RNA sequencing on eWAT. A principal component analysis using the log_2_ transformed expression values shows that the samples from both LFD groups cluster closely together, indicating the absence of a strong Mg^2+^ effect, whereas there is a clear separation of NormMg-HFD vs LowMg-HFD gene expression profiles (Fig. 4e). To investigate the effect of Mg^2+^ on specific biological pathways, the fold changes for groups of genes belonging to the same gene ontology (GO) were analysed. GO term analysis indicated that processes associated with adiposity (e.g. inflammation) were downregulated in LowMg-HFD-fed vs NormMg-HFD-fed mice, in accordance with decreased adipose tissue mass (ESM Table 2). Interestingly, despite the increased insulin sensitivity of the LowMg-HFD-fed mice, several key genes involved in the triacylglycerol catabolism pathway (lipolysis) were upregulated in the LowMg-HFD vs the NormMg-HFD group, which may explain the reduced lipid storage as well as the high serum NEFA levels (Fig. 4f). A modest increase in acyl-CoA dehydrogenase dependent β oxidation was observed in the LowMg-HFD-fed mice vs the NormMg-HFD-fed mice (Fig. 4g). The metabolic effects of Mg^2+^ in eWAT appear to be specific to lipid homeostasis, as there was no clear effect on glycolysis (ESM Fig. 3g). Although serum cholesterol levels were not different between the experimental groups, cholesterol biosynthesis was greatly reduced in the LowMg-HFD-fed vs the NormMg-HFD-fed mice (ESM Fig. 3h, ESM Table 2).

To investigate whether hypomagnesaemia has a direct effect on lipolysis in eWAT, we examined the effect of Mg^2+^ on lipolysis in differentiated 3 T3-L1 cells in vitro. Unstimulated lipolysis was not different at 0 or 1 mmol/l Mg^2+^, indicating that Mg^2+^ deficiency does not directly induce lipolysis in adipocytes (ESM Fig. 4a).

### mRNA expression of the β3-adrenergic receptor is increased in LowMg-HFD mice

**Fig. 5:**
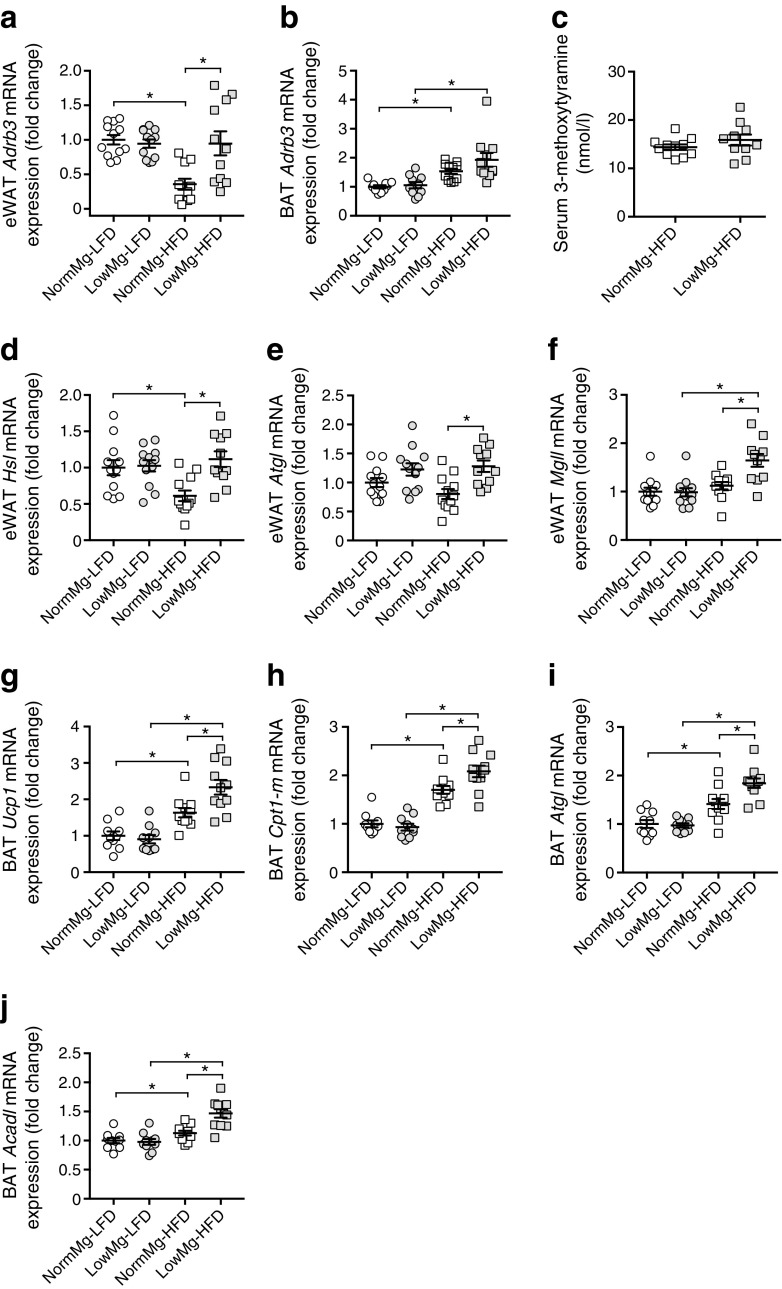
mRNA expression of the β_3_-adrenergic receptor is increased in eWAT of LowMg-HFD-fed mice. Relative mRNA expression of *Adrb3* in (**a**) eWAT and (**b**) BAT, normalised to *Gapdh* expression, relative to NormMg-LFD. (**c**) Serum 3-methoxytyramine concentration at death (17 weeks, *n* = 12 mice in the NormMg-HFD group, *n* = 11 in the LowMg-HFD group). eWAT mRNA expression of genes essential for lipolysis, (**d**) *Hsl*, (**e**) *Atgl* and (**f**) *Mgll*, normalised to *Gapdh* expression, relative to NormMg-LFD (*n* = 12 mice per group, *n* = 11 mice for the LowMg-HFD group). BAT mRNA expression of (**g**) *Ucp1* and genes involved in fatty acid metabolism, (**h**) *Cpt1-m*, (**i**) *Atgl* and (**j**) *Acadl*, normalised to *Gapdh* expression, relative to NormMg-LFD (*n* = 10 mice for both LFD groups, *n* = 11 mice for both HFD groups). NormMg-LFD (white circles), LowMg-LFD (grey circles), NormMg-HFD (white squares), LowMg-HFD (grey squares). Data are mean ± SEM. Depending on the absence or presence of a significant interaction effect between dietary fat and Mg^2+^ content, either a two-way ANOVA (Tukey’s multiple comparison test) or an unpaired multiple *t* test (Holm–Sidak multiple comparison test) approach, respectively, was used to determine statistical significance. Statistical significance in 3-methoxytyramine levels was assessed using a *t* test. *p < 0.05 for the comparisons shown

β_3_-Adrenergic receptors (ADRB3) are essential regulators of lipid metabolism, increasing brown adipose tissue (BAT) activity and reducing WAT lipid storage via activation of lipolysis [29–32]. We therefore explored whether enhanced β-adrenergic signalling could explain the high triacylglycerol levels, increased lipolysis and reduced body weight of Mg^2+^-deficient HFD-fed mice. Expression of *Adrb3* was significantly increased by 2.5-fold in the eWAT of LowMg-HFD-fed compared with NormMg-HFD-fed mice (Fig. 5a). Additionally, both HFD-fed groups showed elevated mRNA expression of *Adrb3* in BAT, but this upregulation was more pronounced in the LowMg-HFD group (Fig. 5b). To determine whether this was the result of enhanced adrenaline (epinephrine) release, serum levels of the dopamine metabolite 3-methoxytyramine and the adrenaline metabolite normetanephrine were measured. However, no significant increase was observed (Fig. 5c and ESM Fig. 5a). mRNA expression of the lipolysis genes adipose triacylglycerol lipase (*Atgl*), hormone-sensitive lipase (*Hsl*) and monoglyceride lipase (*Mgll*) was significantly increased in eWAT of Mg^2+^-deficient HFD-fed mice compared with the NormMg-HFD group (Fig. 5d–f).

Expression of *Ucp1* in BAT, which is essential for non-shivering thermogenesis, was upregulated in NormMg-HFD-fed mice compared with NormMg-LFD-fed mice (Fig. 5g). In line with increased β_3_-adrenergic signalling, *Ucp1* expression was further increased in BAT of LowMg-HFD-fed mice. BAT thermogenesis is strongly regulated by fatty acid availability [33]. Indeed, genes involved in NEFA metabolism of BAT are upregulated (Fig. 5h–j) (*Atgl*, *Cpt1-m* and *Acadl*). In contrast, mRNA levels of glucose transporters 1 and 4 (*Glut1/4*) in BAT were unchanged in LowMg-HFD-fed compared with NormMg-HFD-fed mice (ESM Fig. 5b,c). mRNA expression of the fatty acid transporter *Cd36* and of the important metabolic transcription factors peroxisome proliferator-activated receptor alpha (*Pparα*) and gamma (*Pparγ*) remained unchanged in BAT between the NormMg-HFD-fed and LowMg-HFD-fed mice (ESM Fig. 5d–f).

### LowMg-HFD-fed mice have increased body temperature but equal energy expenditure

**Fig. 6:**
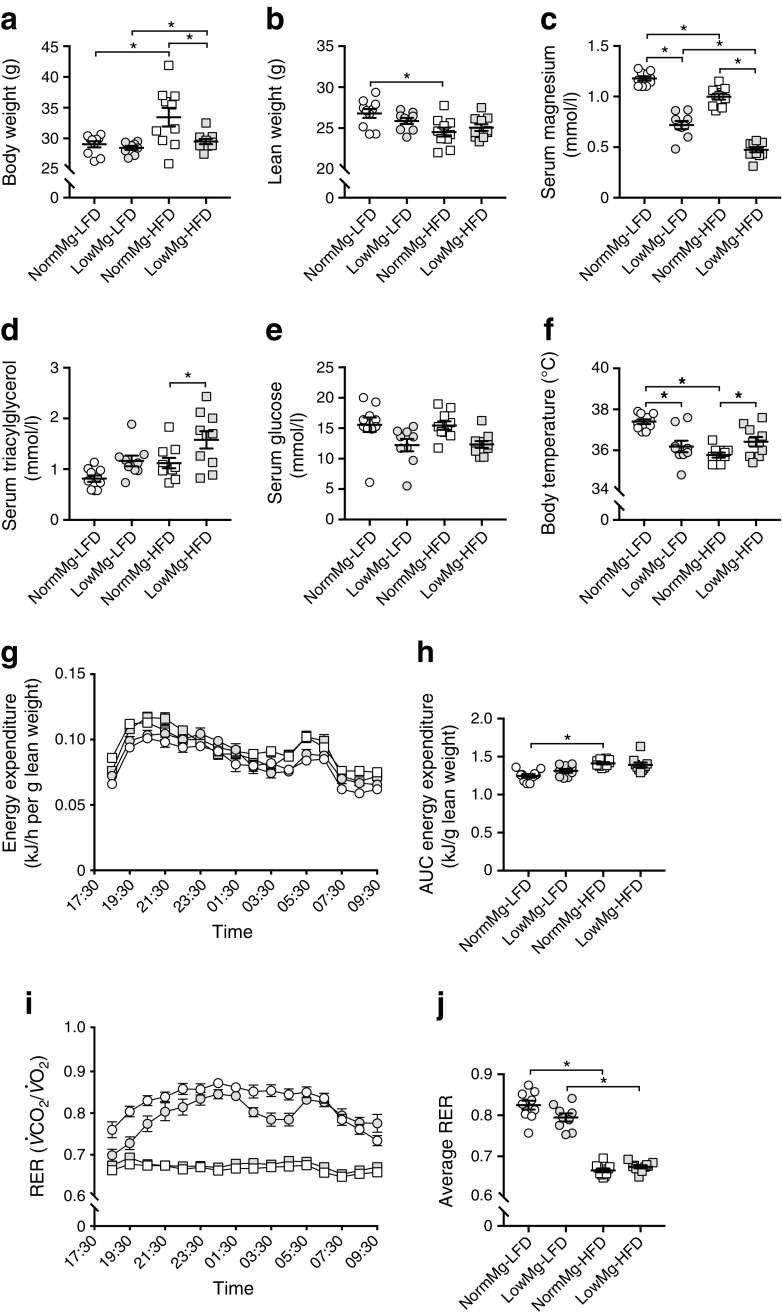
LowMg-HFD-fed mice have increased body temperature but equal energy expenditure. To study energy expenditure, a replication animal study was performed for a duration of 9 weeks. (**a**) Body weight and (**b**) lean body weight of the animals (*n* = 10 mice per group, *n* = 9 mice in the LowMg-LFD group) at death (9 weeks). Non-fasted serum (**c**) Mg^2+^, (**d**) triacylglycerol and (**e**) glucose concentrations at death (glucose at 9 weeks, two-way ANOVA for dietary Mg^2+^ effect *p* < 0.05; NormMg-HFD vs LowMg-HFD Tukey’s test *p* = 0.07). (**f**) Body temperature measured by rectal probe after 8 weeks of dietary intervention. Respiratory metabolic cages were used to determine energy expenditure and RER. (**g**) Energy expenditure averaged per hour, measured after 8 weeks of dietary intervention and corrected for lean weight, (**h**) from which the AUC is calculated. NormMg-LFD (white circles, *n* = 10), LowMg-LFD (grey circles, *n* = 9), NormMg-HFD (white squares, *n* = 10), LowMg-HFD (grey squares, *n* = 10). (**i**) RER averaged per hour, measured after 8 weeks of dietary intervention. RER is determined by dividing the CO_2_ production by the O_2_ intake. (**j**) Average RER over the entire duration of the measurement (from 18:30 to 09:30 h). NormMg-LFD (white circles), LowMg-LFD (grey circles), NormMg-HFD (white squares), LowMg-HFD (grey squares). Data are mean ± SEM. Depending on the absence or presence of a significant interaction effect between dietary fat and Mg^2+^ content, either a two-way ANOVA (Tukey’s multiple comparison test) or an unpaired multiple *t* test (Holm–Sidak multiple comparison test) approach, respectively, was used to determine statistical significance. *p < 0.05 for the comparisons shown

To investigate the energy metabolism in Mg^2+^-deficient HFD-fed mice, the dietary intervention study was repeated with respiratory cages. Respiration, body temperature and activity were measured at week 8, which was when the weight differences developed in our first experiment. In line with the previous experiment, no differences were observed in food and water intake between the two HFD-fed groups (ESM Fig. 6a–d); and the Mg^2+^-deficient HFD-fed mice had reduced body weight compared with the NormMg-HFD-fed mice (Fig. 6a). Lean body mass was not different between the two HFD groups, indicating that the weight difference depends on adipose tissue mass (Fig. 6b). As with our previous experiment, the HFD caused a reduction in serum Mg^2+^ levels (Fig. 6c). A significant increase was observed in serum triacylglycerol when the animals were killed (after 9 weeks) in the LowMg-HFD group compared with the NormMg-HFD group (Fig. 6d). Low dietary Mg^2+^-fed mice had decreased non-fasted serum glucose (Fig. 6e; two-way ANOVA for dietary Mg^2+^ effect *p* < 0.05), while the difference between the two HFD-fed groups was not significant (NormMg-HFD vs LowMg-HFD, Tukey’s test *p* = 0.07). Hypomagnesaemia and HFD decreased core body temperature (Fig. 6f). In contrast, the body temperature of LowMg-HFD-fed mice was higher than NormMg-HFD-fed mice (Fig. 6f; 35.8 ± 0.1 vs 36.4 ± 0.2°C in NormMg-HFD and LowMg-HFD, *p* < 0.05), in line with increased BAT activity. Moreover, mice fed a Mg^2+^-deficient diet showed increased locomotor activity (ESM Fig. 6e,f; two-way ANOVA for dietary Mg^2+^ effect *p* < 0.05). However, energy expenditure was not different between the two HFD-fed groups (Fig. 6g,h). In both HFD groups, the respiratory exchange ratio (RER) was approximately 0.7, indicating that fatty acids are the main energy source (Fig. 6i,j). Interestingly, in the LFD groups, hypomagnesaemia resulted in a reduction of the RER (Fig. 6i,j), suggesting an increased use of fatty acids as an energy substrate over glucose. However, this reduction was not statistically significant (Fig. 6j; 0.82 ± 0.01 vs 0.79 ± 0.01 average RER in NormMg-LFD and LowMg-LFD, respectively; *p* = 0.10).

## Discussion

Hypomagnesaemia has been repeatedly reported in type 2 diabetes and the metabolic syndrome [1, 2, 14], but the role of Mg^2+^ in lipid metabolism has been largely overlooked. Here, we demonstrate that low dietary Mg^2+^ intake ameliorates HFD-induced obesity. The lower body weight results in beneficial metabolic effects including improved insulin sensitivity, reduced hepatic steatosis and lower WAT inflammation. Nevertheless, serum triacylglycerol and NEFA concentrations were increased in the low Mg^2+^ HFD group, corresponding to increased eWAT mRNA expression of lipolysis genes. These findings establish Mg^2+^ as an important regulator of body weight and lipid metabolism.

In this study, a Mg^2+^-deficient diet ameliorated HFD-induced weight gain in mice. This was the result of reduced adiposity, because lean body mass was similar between the two HFD groups and both eWAT and iWAT weight were lower in mice fed a LowMg-HFD compared with a NormMg-HFD. The reduced body weight was associated with favourable metabolic effects. IPGTT, IPITT and fasting glucose levels indicated enhanced insulin sensitivity. Moreover, the reduced body weight of the LowMg-HFD mice led to a complete absence of hepatic steatosis and RNA sequencing of the eWAT demonstrated downregulation of pro-inflammatory pathways. Despite these beneficial effects, blood lipid levels remained high. In line with our data, others have demonstrated that low dietary Mg^2+^ intake reduced body weight in several rat models of Mg^2+^ deficiency [34–37]. However, these studies did not address the underlying cause or investigate the effects on lipid metabolism.

Our animal data is strengthened by the results of Chubanov et al [17] where severe hypomagnesaemia via *Trpm6* knockout also resulted in a catabolic phenotype and improved insulin sensitivity [17]. The catabolic phenotype of Mg^2+^-deficient mice leads to hyperlipidaemia, which has considerable adverse effects in individuals with type 2 diabetes [38, 39]. Nevertheless, the low Mg^2+^ HFD does not completely mimic the human situation because the hypomagnesaemia induced in mice is more severe [1]. Moreover, an unhealthy human diet consists of both high fat and sugar, whereas the HFD in mice purely depends on palm oil. Indeed, Mg^2+^-deficiency in high-fructose diets has adversely affected insulin sensitivity and lipid homeostasis in rats. This shows the considerable differences in the role of Mg^2+^ in the metabolism of lipids vs carbohydrates [40, 41]. Future studies should investigate the role of Mg^2+^ in combined fat and sugar diets. These differences may explain why, in humans, higher oral Mg^2+^ intake and Mg^2+^ supplementation have beneficial effects on metabolic variables, which apparently contrasts with our animal data [18–20].

In our study, the reduced WAT mass of LowMg-HFD-fed mice was associated with enhanced lipolysis gene expression, causing high serum NEFA and triacylglycerol levels. These findings suggest that LowMg-HFD-fed mice depend more on mitochondrial β-oxidation, rather than glycolysis, for energy production. However, our energy metabolism experiments demonstrated neither differences in energy expenditure nor in RER between the NormMg-HFD and LowMg-HFD groups. It should be noted, however, that both HFD groups mainly depend on lipids for energy metabolism, masking potential RER differences between these groups. Moreover, despite equal energy expenditure, the NormMg-HFD-fed mice are considerably heavier than LowMg-HFD-fed mice and therefore have a higher energy demand. Several studies have discussed the considerable difficulties associated with the interpretation of energy expenditure data and emphasised that body weight differences complicate interpretation [42, 43]. Increased thermogenesis may explain why energy expenditure does not differ between LowMg-HFD-fed and the heavier NormMg-HFD-fed mice. Although the effects are modest, the LowMg-HFD-fed mice had a significantly higher body temperature and increased *Ucp1* expression in BAT, indicative of higher thermogenesis. Cold-exposure studies are necessary to further investigate the role of Mg^2+^ status in BAT activation, WAT browning and thermogenesis.

The increased lipolysis and brown adipose tissue activity were associated with higher β_3_-adrenergic receptor expression in eWAT and BAT of LowMg-HFD-fed mice. β_3_-receptor knockout mice have increased lipid stores and impaired WAT browning [44, 45]. Activation of the β_3_-adrenergic receptors in mice using agonist CL316243 decreases adipose tissue mass, improves insulin sensitivity, increases uncoupling protein-1 (UCP1)-dependent thermogenesis and activates a cycle of concomitant lipolysis and de novo lipogenesis [46, 47]. Interestingly, this is exactly the phenotype that was observed in the LowMg-HFD-fed mice, although to a lesser extent. A link between Mg^2+^, β-adrenergic signalling and lipolysis is not without precedent. Use of β-adrenergic agonists, which stimulate lipolysis, have been associated with decreased blood Mg^2+^ levels [1, 48, 49]. Mg^2+^ has also been shown to reduce catecholamine release from the adrenal medulla [50] and Mg^2+^ deficiency is associated with higher urinary levels of adrenaline and noradrenaline (norepinephrine) [37]. Moreover, Mg^2+^ supplementation has been suggested to regulate lipolysis, as it prevents hyperlipidaemia in diabetic rats and reduces serum triacylglycerol levels in individuals with type 2 diabetes [20, 51]. Further research is required to determine exactly how hypomagnesaemia increases β-adrenergic signalling and how β-adrenergic signalling can induce hypomagnesaemia.

A strength of this study is that the model used to induce type 2 diabetes and low dietary Mg^2+^ intake closely resembles the human situation. The Western diet contains high amounts of processed foods consisting of high energy and low Mg^2+^. Moreover, the extensive phenotyping of the animals in this study provides new avenues for research into the pivotal role of Mg^2+^ in metabolism. The data obtained in this study are robust, as a replicate animal experiment was performed in a separate institution, confirming our results.

Our study has limitations. First, because of the large weight differences induced by the Mg^2+^ deficient diet, it is difficult to specifically attribute the metabolic changes of the mice to their lower body weight or their Mg^2+^ deficiency. In addition, our study design did not allow us to study in more depth the contribution of disturbed β-adrenergic signalling to the differences in body weight, eWAT lipolysis, BAT activity and hyperlipidaemia. Although our data and previous studies support a role for Mg^2+^ in β-adrenergic signalling [37, 50], further studies are required to establish the exact role of Mg^2+^ in catecholamine secretion and signalling.

In conclusion, our results demonstrate that hypomagnesaemia in mice prevents HFD-induced weight gain by enhanced BAT activity and increased eWAT lipolysis gene expression. Consequently, this led to improved insulin sensitivity and absent hepatic steatosis. These results underline the pivotal function of Mg^2+^ in maintaining a healthy energy metabolism.

## Electronic supplementary material

ESM (PDF 2710 kb)

## Data Availability

The data and materials that support the findings of this study are available from the corresponding author upon reasonable request. The RNA sequencing data have been submitted to the gene expression omnibus (GEO) database (accession no. GSE116270).

## Abbreviations

BAT: Brown adipose tissue
eWAT: Epididymal white adipose tissue
HFD: High-fat diet
GO: Gene ontology
iWAT: Inguinal white adipose tissue
LFD: Low-fat diet
LowMg: Low Mg^2+^ (diet)
NormMg: Normal Mg^2+^ (diet)
RER: Respiratory exchange ratio
WAT: White adipose tissue
