# Supplementary material for: Magnesium deficiency prevents high-fat-diet-induced obesity in mice
Source: Diabetologia. 2018 Jul 9;61(9):2030–42. doi: 10.1007/s00125-018-4680-5 (PMC6096631; doi:10.1007/s00125-018-4680-5)
Supplement: Supplementary file 1 — (PDF 2710 kb) [file 125_2018_4680_MOESM1_ESM.pdf]

ELECTRONIC SUPPLEMENTARY MATERIAL (ESM) METHODS

17-week mouse study – Radboud university medical center

This study was approved by the animal ethics board of the Radboud University Nijmegen (RU DEC 2015-0073) and the Dutch Central Commission for Animal Experiments (CCD, AVD103002015239). To study the effect of dietary Mg^{2+} on HFD-induced obesity, 48 male C57BL6/J mice (Charles River, Germany), age 9-10 weeks, were randomly allocated into four experimental groups of n=12 mice. Mice were acclimatized for two weeks in a temperature- and light-controlled room, six per cage (Eurostandard Type III), and had *ad libitum* access to acidified tap water and standard pellet chow (Ssniff Spezialdiäten, GmbH, Germany). Experimental diets consisted of both 10 or 60 kcal% palm oil and 0.03 or 0.21 w/w% magnesiumoxide (Ssniff Spezialdiäten, NormalMg-LFD #S9074-E0277, LowMg-LFD #S9074-E0287, NormalMg-HFD #S9074-E0297, LowMg-HFD #S9074-E0317). Researchers and animal caretakers were blinded for the Mg^{2+} content of the experimental diets throughout the experiment. At day -1, 84 and 112, mice were housed individually in metabolic cages for 24 hours for the collection of urine and faeces and determining food and water intake. Mice were weighed twice weekly and blood was collected *via* cheek puncture at day -1, 28, 56 and 84. At week 14 and 15 insulin and glucose tolerance tests, respectively, were performed. One mouse died unrelated to the dietary intervention and was excluded from future analyses. After 17 weeks on the diets, mice were anaesthetized by 4 v/v% isoflurane and exsanguinated *via* orbital sinus bleeding. Death was confirmed by cervical dislocation. Tissues were stored in 10 v/v% formalin or snap frozen in liquid nitrogen.

Intraperitoneal insulin and glucose tolerance tests

After 14 weeks on the diets, three mice per group per day underwent an intraperitoneal insulin tolerance test (IPITT), over a period of four days (n=9 mice/group). After 6 hours of fasting from 08:00 AM, mice were injected with 0.75 U/kg bodyweight human insulin (Novorapid, Novo Nordisk, Denmark) dissolved in phosphate buffered saline (PBS). Blood glucose levels were measured at 0, 20, 40, 60, 90 and 120 minutes after the insulin injection. Blood was taken *via* a tail cut and glucose levels were measured using a StatStrip® Xpress glucose meter (Nova Biomedical, USA). Three mice developed severe hypoglycaemia and

were injected with D-glucose (Invitrogen, The Netherlands) between 15 and 30 minutes post insulin-injection. For the subsequent time points, these mice were given the same glucose values as the lowest values within that group. After 15 weeks on the diets, three mice per group per day underwent an intraperitoneal glucose tolerance test (IPGTT), over a period of four days (n=12 mice/group). After an overnight fast (from 18:00 to 09:00), mice were injected with 2 g/kg body weight D-glucose (Invitrogen, The Netherlands) dissolved in PBS, with an injection volume of 50ul/10g body weight. Blood glucose levels were measured at 0, 15, 30, 60 and 120 minutes after the glucose injection.

RNA-Sequencing

Total RNA was extracted from epididymal white adipose tissue using TRIzol reagent (Invitrogen, UK) according to manufacturer's protocol. RNA integrity was validated by investigating the 18S/28S bands on a 2 w/v% agarose gel. Five randomly selected samples of each group were RNA-sequenced (RNA-Seq). Quality control and RNA-Seq were performed by Beijing Genomics Institute (BGI), Hong Kong). Quality control was performed by using Agilent 2100 Bioanalyzer and ABI StepOnePlus Real-Time PCR System to qualify and quantify the sample library. One sample from the NormalMg-LFD group failed the quality control, and was excluded from subsequent sequencing and analyses. One sample from the LowMg-HFD group showed a small contamination with pancreatic tissue and was excluded from subsequent analyses. 13 million reads were sequenced using the Hiseq 4000 platform (Illumina, USA) using a 50 bp single-end module. Clean reads were mapped to Mus Musculus transcriptome (GRCm38/mm10) using HISAT/Bowtie2 tool [24, 25]. RSEM software v1.2.31 was used to quantify gene expression levels (FPKM values)[26]. FPKM values were $\log_2$ transformed and further analysed in R (www.r-project.org, RRID:SCR_001905). In order to filter non-expressed transcripts from the data, only transcripts that showed an average expression level of 8 within a group and for which the transcript levels were above 8 in at least four replicates from an experimental group were retained, yielding a total of 8808 transcripts. To calculate the differences between expression levels for genes belonging to the same Gene Ontology group, the fold change between the LowMg and NormalMg condition for both the HFD and LFD groups were collected for each gene in the group. Subsequently a *t*-test was used to test for the hypothesis of equal means. The procedure was repeated for all

GO terms and the *p*-values for the tests were corrected for multiple testing using the Benjamini-Hochberg method as implemented in the p.adjust method in R. Heatmaps for individual GO terms were created using the ggplot2 library (RRID:SCR_014601) [27].

9-Week replication mouse study – MRC Harwell Institute

All experimental procedures were conducted in compliance with the UK Animals Scientific Procedures Act (1986) and University of Oxford ethical guidelines. 39 male C57BL6/J mice (MRC Harwell, UK) were randomly allocated into 4 groups of n=10 mice (n=9 in the LowMg-LFD group) housed with five per cage (1284L and 1285L IVC, Tecniplast, Italy). Mice had *ad libitum* access to demineralized chlorinated tap water and standard pellet chow. At 8 weeks old, mice were put on experimental diets identical to the first animal experiment at the Radboudumc, for a period of 9 weeks. At day 14, mice were housed individually in metabolic cages (Tecniplast, Italy) for 24 hours for the collection of urine and faeces and determining food and water intake. Mice were weighed twice weekly and blood was collected *via* tail bleed at day -1 and 14. Respiration metabolic cages (TSE Phenomaster Cages, Germany) were used at day 28 and 56 of the experiment and body temperatures were measured by rectal probe (ATP-instrumentation, UK). Data were averaged per hour and plotted from 6:30 PM to 9:30 AM. After 9 weeks on the diets, mice were anaesthetized by 4 v/v% isoflurane and exsanguinated *via* orbital sinus bleeding. Death was confirmed by cervical dislocation. Tissues were stored in 10 v/v% formalin or snap frozen in liquid nitrogen.

Lipolysis in 3T3-L1 adipocytes

3T3-L1 fibroblasts (ATCC, mycoplasma-free) were cultured in Dulbecco's modified Eagle's medium (DMEM, Lonza Westburg, Leusden, The Netherlands) containing 2 mg/ml ciproxin (Fresenius Kabi, Zeist, The Netherlands), 200 mmol/l L-glutamine (GE healthcare Life Sciences, Logan, UT, USA) and 10 v/v% fetal bovine serum (FBS, Greiner Bio One), at 37 °C, in 5 v/v% CO_2. 3T3-L1 cells between passages 10 and 20 were differentiated according to ATCC's protocol. In short, cells were seeded in PLL (Sigma-Aldrich) coated 6-well plates in DMEM and upon confluence medium was refreshed. Two days post-confluence the induction process was initiated by changing the medium to induction medium containing 1 µg/ml bovine

insulin (Sigma-Aldrich, St. Louis, MO, USA), 0.5 mmol/l IBMX (Sigma-Aldrich), and 1 μmol/l dexamethasone (Sigma-Aldrich) for 48 hours. The cells were then washed (PBS) and DMEM containing 1 μg/ml insulin was added. Hereafter, the medium was refreshed every two days until >90% of the cells were completely differentiated into adipocytes. To determine the effect of Mg^{2+} on lipolysis, differentiated 3T3-L1 cells were incubated for 20 hours in DMEM without added insulin, containing 1.0 or 0 mmol/l $MgCl_2$, followed by 2 hours serum-starvation. Hereafter, cells were incubated for 4 hours in 700 μl KRPH buffer (20 mmol/l HEPES pH 7.4, 5 mmol/l KH_2PO_4, 1 mmol/l $CaCl_2$, 136 mmol/l NaCl, 4.7 mmol/l KCl at 37 °C) containing 1.0 or 0 mmol/l $MgCl_2$, 0.1 w/v% glucose (Merck Millipore, Amsterdam, The Netherlands) and 3.5 w/v% fatty acid free BSA (Sigma-Aldrich). 50 μl aliquots of the medium were taken every hour and heated for 8 minutes at 65 °C. The concentration of NEFAs was assessed using the WAKO NEFA-C kit (Instruchemie, Delfzijl, The Netherlands) according to the manufacturer's protocol.

S1

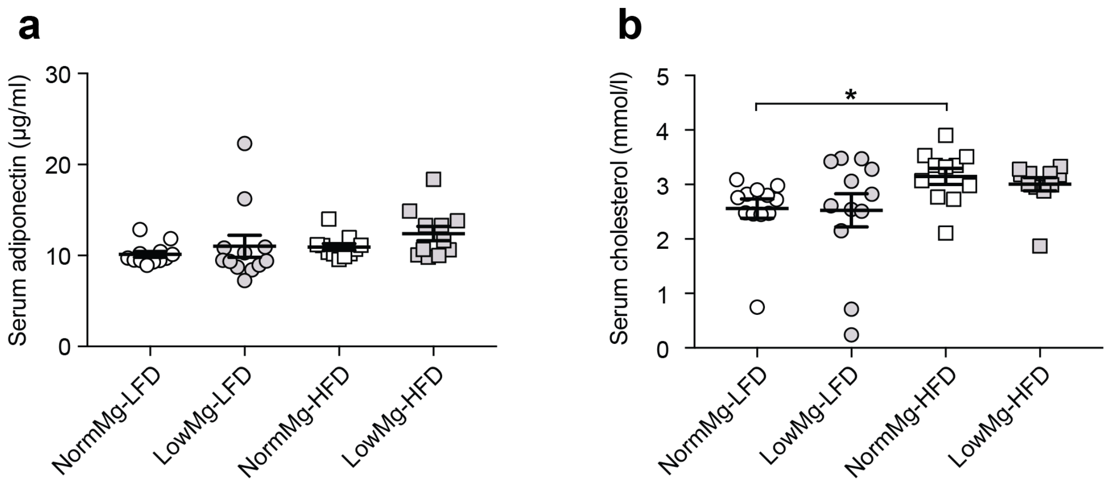

ESM Fig. 1. Changes in dietary Mg^{2+} or fat content do not affect serum adiponectin and cholesterol concentrations. Non-fasted serum concentrations of (a) adiponectin and (b) cholesterol at killing (n=12 mice for both LFD groups, n=11 mice for both HFD groups).

Symbols: NormalMg-LFD (white circle), LowMg-LFD (light grey circle), NormalMg-HFD (white square), LowMg-HFD (light grey square). Data are mean ± SEM. Depending on the absence or presence of a significant interaction effect between dietary fat and Mg^{2+} content, either a two-way ANOVA (Tukey's multiple comparison test) or a multiple t-test (Holm-Sidak multiple comparison test) approach, respectively, was used to determine statistical significance. * Indicates $p<0.05$.

S2

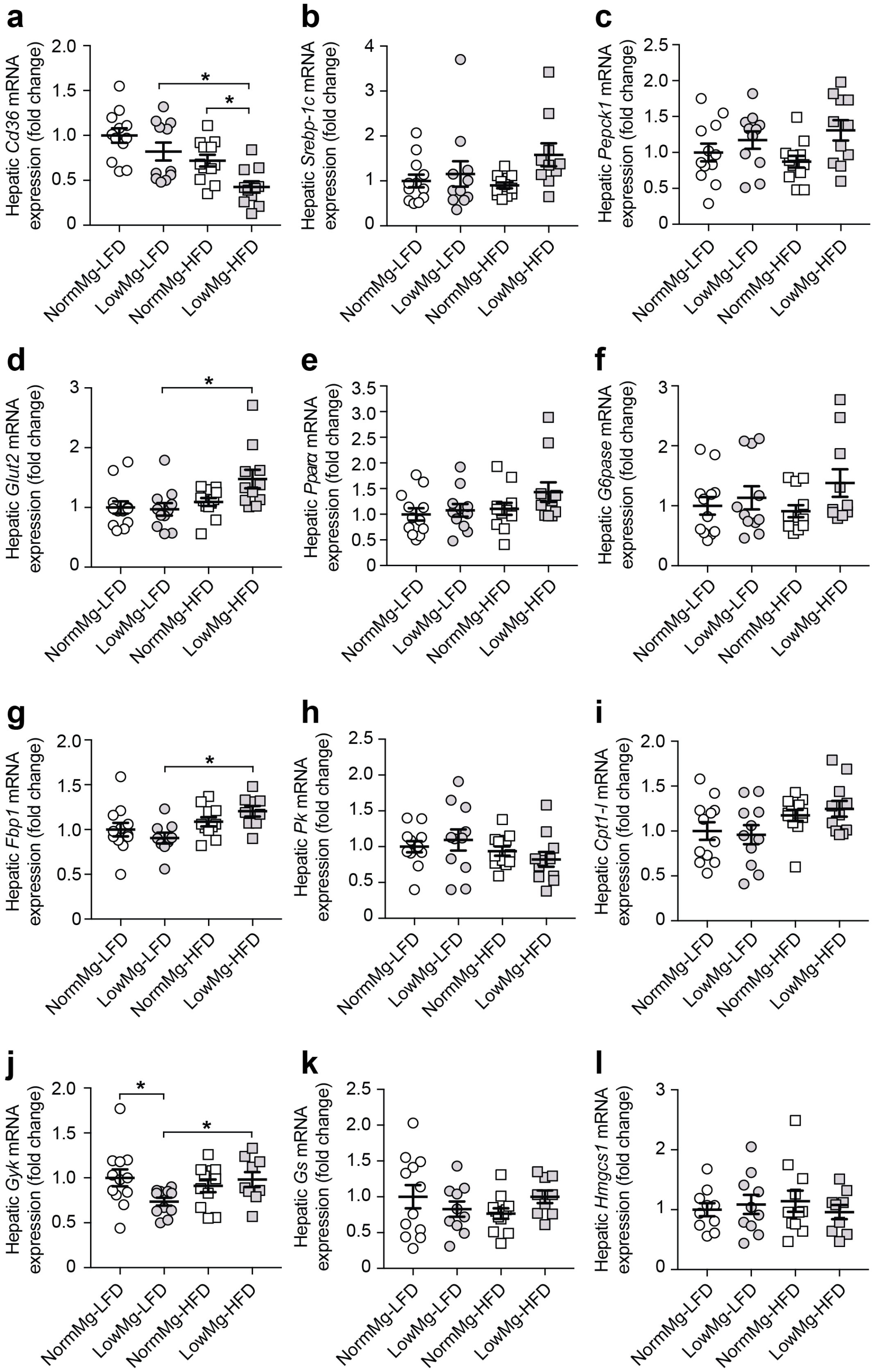

ESM Fig. 2. Hepatic mRNA expression of key genes involved in energy metabolism. Hepatic mRNA expression of (a) *Cd36,* (b) *Srebp1c* (two-way ANOVA for dietary Mg^{2+} effect $p<0.05$), (c) *Pepck1* (two-way ANOVA for dietary Mg^{2+} effect $p<0.05$; NormMg-HFD vs.

LowMgHFD Tukey's test *p*=0.06), (d) *Glut2,* (e) *Pparα,* (f) *G6pase*, (g) *Fbp1*, (h) *Pk-lr*, (i) *Cpt1-l* (two-way ANOVA dietary fat effect *p*<0.05), (j) *Gyk*, (k) *Gs* and (l) *Hmgcs1* normalized to *Gapdh* expression, relative to NormalMg-LFD (n= 12 mice per group, n=11 for the LowMg-HFD group).

Symbols: NormalMg-LFD (white circle), LowMg-LFD (light grey circle), NormalMg-HFD (white square), LowMg-HFD (light grey square). Data are mean ± SEM. Depending on the absence or presence of a significant interaction effect between dietary fat and Mg^{2+} content, either a two-way ANOVA (Tukey's multiple comparison test) or a multiple t-test (Holm-Sidak multiple comparison test) approach, respectively, was used to determine statistical significance. * Indicates *p*<0.05.

Cd36, Cluster of differentiation 36; *Srebp1*, Sterol regulatory element-binding protein; *Pparα*, Peroxisome proliferator-activated alpha; *Glut2,* Glucose transporter 2; *Pepck1*, Phosphoenoylpyruvate carboxykinase 1; *G6pase*, Glucose-6-phosphatase; *Fbp1*, Fructose-1,6-bisphosphatase 1; *Pk-lr*, Pyruvate kinase liver and RBC; *Cpt1-l*, Carnitine palmitoyltransferase 1 liver type; *Gyk,* Glycerol Kinase; *Gs*, Glycogen synthase; *Hmgcs1*, Hydroxymethylglutaryl-CoA synthase 1.

S3

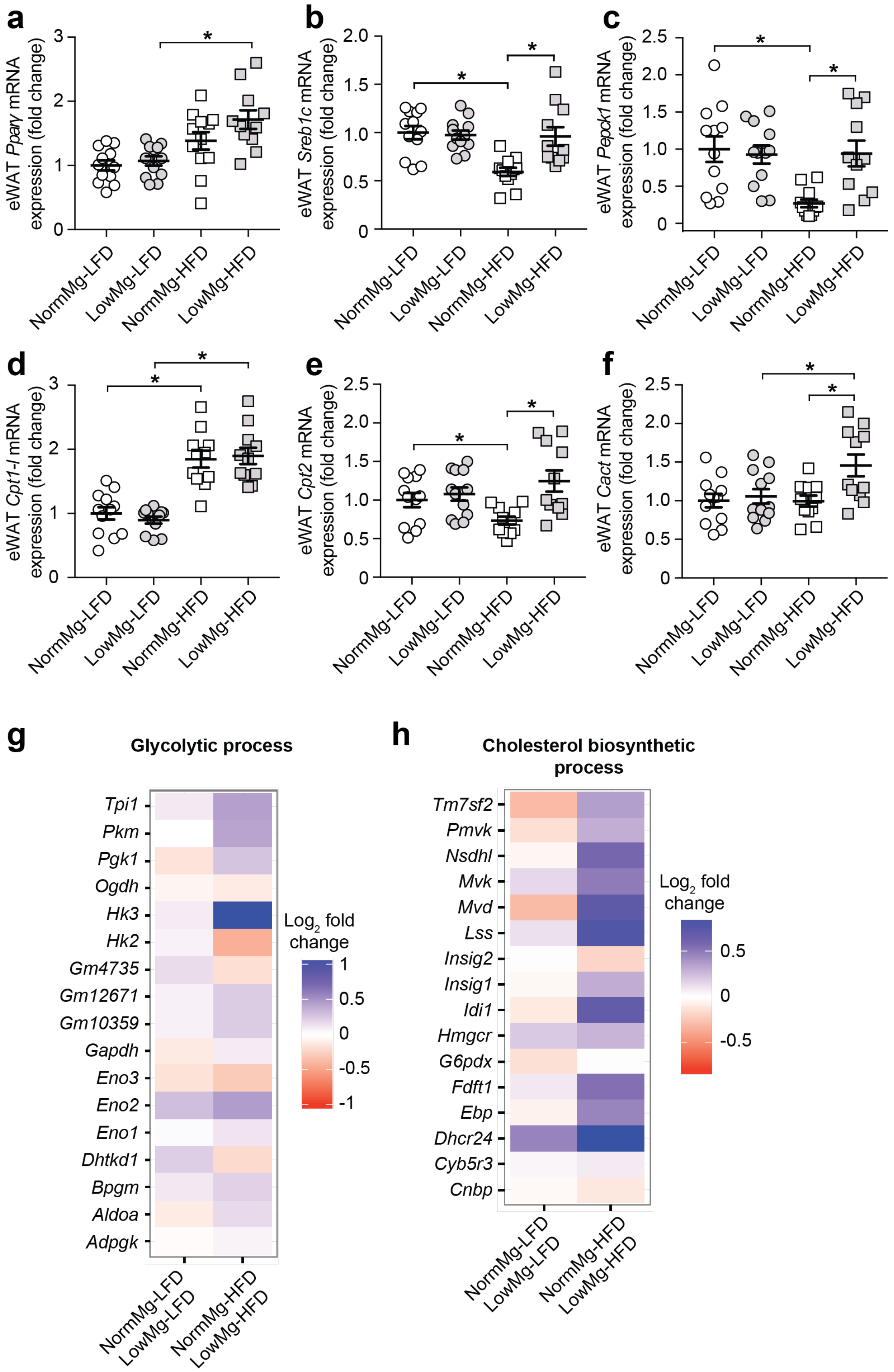

ESM Fig. 3. Increased mRNA expression of key genes involved in gluconeogenesis and beta-oxidation in the eWAT of LowMg-HFD mice and heatmaps of eWAT RNA-Seq GO-Term

glycolysis and cholesterol biosynthesis pathways. Epididymal white adipose tissue (eWAT) mRNA expression of (a) *Pparγ*, (b) *Srebp1c*, (c) *Pepck1*, (d) *Cpt1-l*, (e) *Cpt2* and (f) *Cact*, normalized to *Gapdh* expression, relative to NormalMg-LFD (n= 12 mice per group, n=11 for the LowMg-HFD group). GO-Term Analyses of the Pathways (g) 'Glycolytic Process' and (h) 'Cholesterol Biosynthesis'. Gene expression changes are presented as $\log_2$ fold changes with the NormalMg^{2+} diet as reference, so that a negative value (in red) indicates a decrease in expression in the NormalMg^{2+} versus LowMg^{2+} groups (n= 4 mice for the NormalMg-LFD and LowMg-HFD groups, n=5 for the LowMg-LFD and NormalMg-HFD groups).

Symbols: NormalMg-LFD (white circle), LowMg-LFD (light grey circle), NormalMg-HFD (white square), LowMg-HFD (light grey square). Data are mean ± SEM. Depending on the absence or presence of a significant interaction effect between dietary fat and Mg^{2+} content, either a two-way ANOVA (Tukey's multiple comparison test) or a multiple t-test (Holm-Sidak multiple comparison test) approach, respectively, was used to determine statistical significance. * Indicates $p<0.05$.

Pparγ, Peroxisome proliferator-activated receptor gamma; *Srebp1*, Sterol regulatory element-binding protein; *Pepck1*, Phosphoenoylpyruvate carboxykinase 1; *Cpt1-l*, Carnitine palmitoyltransferase 1 liver type; *Cpt2*, Carnitine palmitoyltransferase 2; *Cact*, Carnitine acyl-carnitine transferase; *Gapdh,* Glyceraldehyde 3-phosphate dehydrogenase.

S4

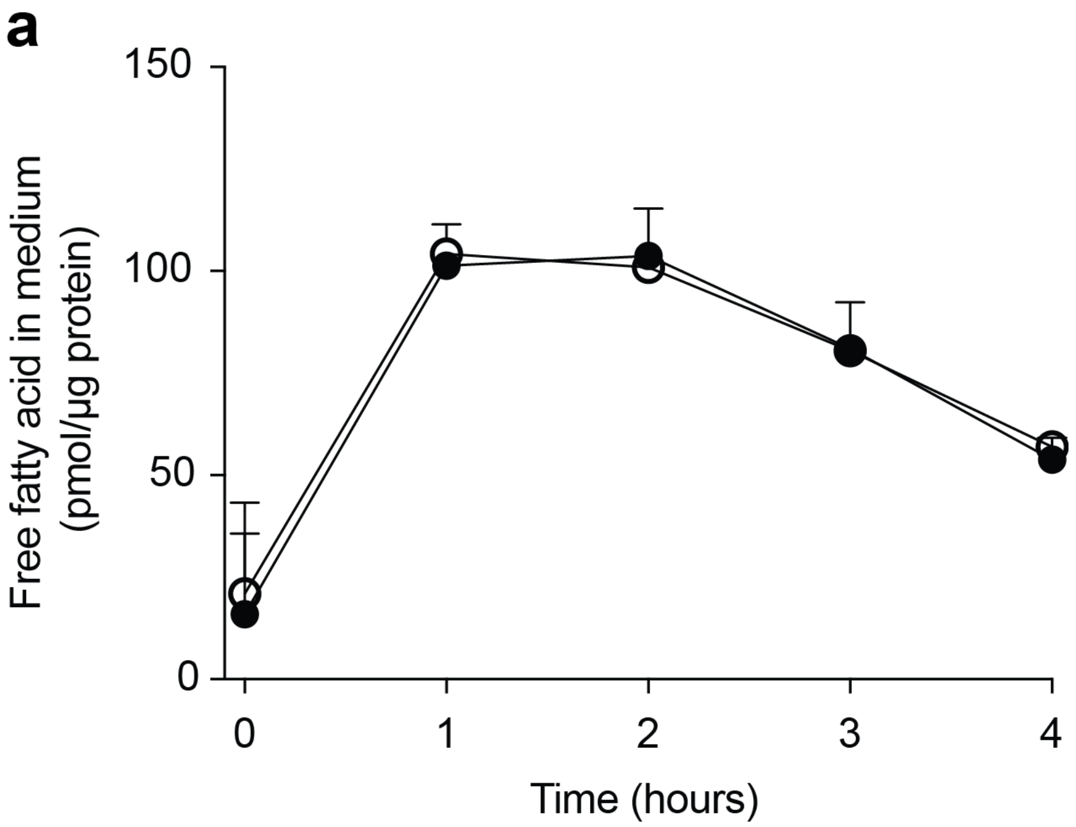

ESM Fig. 4. Extracellular Mg^{2+} deficiency does not directly induce lipolysis in 3T3-1L adipocytes. (a) 3T3-1L cells were differentiated into adipocytes and cultured in 0 (○) or 1 (●) mmol/l extracellular $MgCl_2$ for 22 hours. As a measure of the rate of lipolysis, the non-esterified concentration in the medium was measured over a period of 4 hours (n=3 wells per time point per condition). A figure of a representative experiment is shown. The experiment was repeated with similar results.

Statistical significance was evaluated using a two-tailed Student's t-test. Data mean ± SEM.

S5

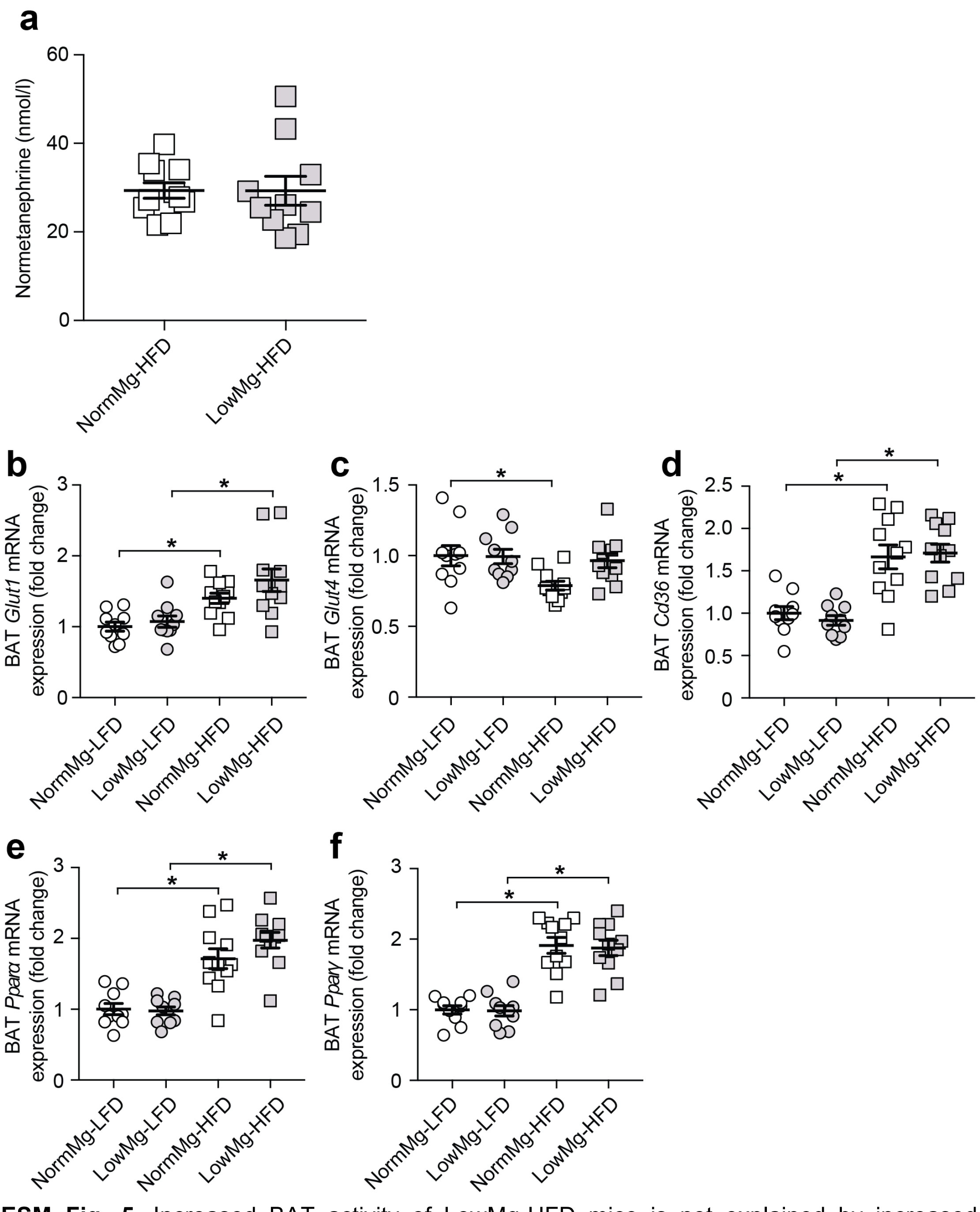

ESM Fig. 5. Increased BAT activity of LowMg-HFD mice is not explained by increased glucose transporter expression or increased PPAR-signalling. (a) Non-fasted serum normetanephrine concentrations (n=11 NormMg-HFD, n=10 LowMg-HFD). Gene expression levels in brown adipose tissue (BAT) on genes involved in energy consumption. BAT mRNA expression of (b) *Glut1*, (c) *Glut4*, (d) *Cd36*, (e) *Pparα* and (f) *Pparγ*, normalized to *Gapdh* expression, relative to NormalMg-LFD (n=10 mice for both LFD groups, n=11 for both HFD groups).

Symbols: NormalMg-LFD (white circle), LowMg-LFD (light grey circle), NormalMg-HFD (white square), LowMg-HFD (light grey square). Data are mean ± SEM. Depending on the absence or presence of a significant interaction effect between dietary fat and Mg^{2+} content, either a two-way ANOVA (Tukey's multiple comparison test) or a multiple t-test (Holm-Sidak multiple

comparison test) approach, respectively, was used to determine statistical significance. Significance in normetanephrin levels was assessed using a t-test. * Indicates $p<0.05$.

Glut, Glucose transporter; *Cd36*, Cluster of differentiation; *Ppar*, Peroxisome proliferator-activated; *Gapdh,* Glyceraldehyde 3-phosphate dehydrogenase.

S6

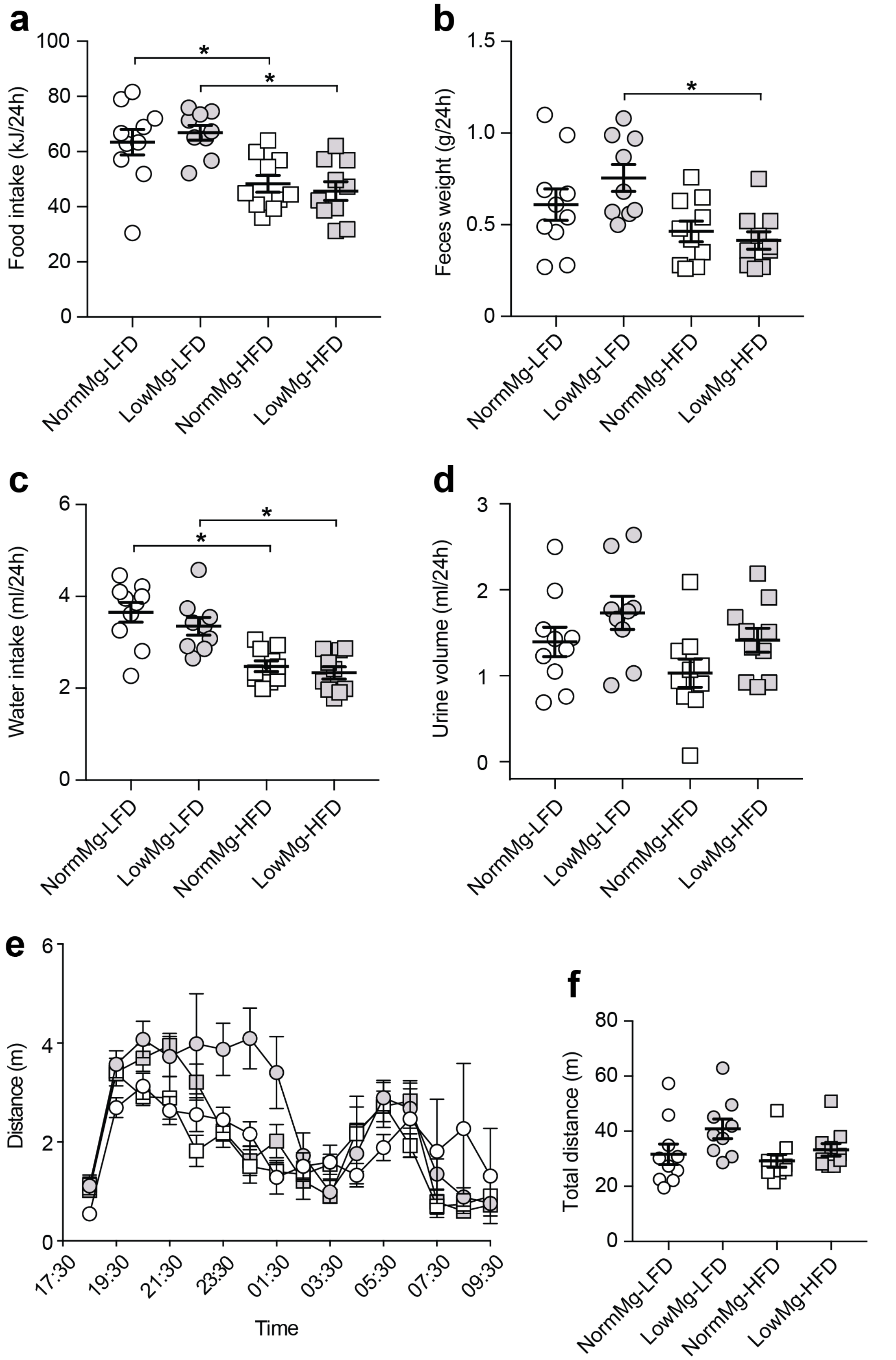

ESM Fig. 6. Dietary Mg^{2+} content does not affect food or water intake after two weeks on the experimental diets. Metabolic cage data from a replication animal study which was performed for a duration of 9 weeks. (a) Food intake, (b) total faeces weight, (c) water intake and (d)

urinary volume (two-way ANOVA for dietary fat and Mg^{2+} effect $p<0.05$) determined over a period of 24 hours, using metabolic cages, at week 2 (n=10 mice per group, n=9 mice in the LowMg-LFD group). (e) Distance walked by mice, averaged per hour from 18:30 to 09:30, measured after 8 weeks of dietary intervention, (f) from which the total distance walked during this period is calculated (two-way ANOVA for dietary Mg^{2+} effect $p<0.05$).

Symbols: NormalMg-LFD (white circle), LowMg-LFD (light grey circle), NormalMg-HFD (white square), LowMg-HFD (light grey square). Data are mean ± SEM. Depending on the absence or presence of a significant interaction effect between dietary fat and Mg^{2+} content, either a two-way ANOVA (Tukey's multiple comparison test) or a multiple t-test (Holm-Sidak multiple comparison test) approach, respectively, was used to determine statistical significance. * Indicates $p<0.05$.

ESM Table 1. RT-qPCR Primer sequences.

Acadl, Acyl-coa dehydrogenase, long chain; *Adrb3,* Beta-3 adrenergic receptor; *Atgl,* Adipose triacylglycerol lipase; *Cact*, Carnitine acyl-carnitine transferase; *Cd36*, Cluster of differentiation; *Cpt1-l*, Carnitine palmitoyltransferase 1 liver type; *Cpt1-m*, Carnitine palmitoyltransferase 1 muscle type; *Cpt2*, Carnitine palmitoyltransferase 2; *Fbp1*, Fructose-1,6-bisphosphatase 1; *G6pase*, Glucose-6-phosphatase; *Gapdh,* Glyceraldehyde 3-phosphate dehydrogenase; *Glut,* Glucose transporter; *Gs*, Glycogen synthase; *Gyk,* Glycerol Kinase; *Hmgcs1*, Hydroxymethylglutaryl-CoA synthase 1; *Hsl,* Hormone-sensitive lipase; *Mgll*, monoacylglycerol lipase; *Pepck1*, Phosphoenoylpyruvate carboxykinase 1; *Pk-lr*, Pyruvate kinase liver and RBC; *Ppar,* Peroxisome proliferator-activated receptor; *Srebp1*, Sterol regulatory element-binding protein; *Ucp1,* Uncoupling protein 1.

Gene	Forward primer (5’ → 3’)	Reverse primer (5’ → 3’)
Acadl	AAACGTCTGGACTCCGGTTC	ACGTAAGCTTTTGCAATCGGG
Adrb3	TCCGTCGTCTTCTGTGTAGC	GCCATCAAACCTGTTGAGCG
Atgl	GAGGAATGGCCTACTGAACCAAC	AGGCTGCAATTGATCCTCCTC
Cact	CGATTCCAGACTGCACCTCC	CGCGGATCATGACTGCATTG
Cd36	TGCATGAATTAGAACCGGGC	TCTCCTCGTGCAGCAGAATC
Cpt1-l	GTGAGCCTGGCCTCGCC	TGAGTGGTGACCGAGTCTGC
Cpt1-m	CTGGGCTATCTGTGTCCGTC	GGGACAGGAAGCTTAGGCAG
Cpt2	GTATCTGCAGCACAGCATCG	GTTTAGGGATAGGCAGCCTGG
Fbp1	CCCAGCTGCTGAATTCGCTC	AGCGATACCATAGAGCTGTGC
G6pase	TTGGACAACGCCCGTATTGG	GGACTTCCTGGTCCGGTCTC
Gapdh	TAACATCAAATGGGGTGAGG	GGTTCACACCCATCACAAAC
Glut1	GGGTCTTAAGTGCGTCAGGG	TCACCTTCTTGCTGCTGGG
Glut2	AGAAGACAAGATCACCGGAACC	TCACACCGATGTCATAGCCG
Glut4	CTTATTGCAGCGCCTGAGTC	GTTCCCCATCGTCAGAGCC
Gs	TGACTGAGCTCAAACGAAATGATTC	TGCATCAGGGTGTGGATCTG
Gyk	GTTGTCCCCTCTGGCTCTTC	AGGCCTGTCTTGGACTTGAC
Hmgcs1	TCCCCTTTGGCTCTTTCACC	TCCCACATCTTTTGGCCAGC
Hsl	AGGGAGGGCCTCAGCG	TGTCTTCTGCGAGTGTCACC
Mgll	CGGAACAAGTCGGAGGGTTC	TGTTTTGTCCTGACTCCGGG
Pepck1	CCTAGTGCCTGTGGGAAGAC	AGCCCTTAAGTTGCCTTGGG
Pk-lr	CAGTATGGAAGGGCCAGCAG	CAGGAAGGTGTCCGCCATAG
Ppar-α	GTGGTGCATTTGGGCGTATC	TGAACTTCAACTTGGCTCTCC
Ppar-γ	CTGACGGGGTCTCGGTTG	CAACCATGGTAATTTCAGTAAAGGGCC
Srebp1	CAGCCACACTTCATCAAGGC	ACTCACCAGGGTCTGC
Ucp1	TGGCCTCTTTAACCCTGCTG	GATTAGGGGTCGTCCCTTTCC

ESM Table 2. List of Differentially Regulated GO-Terms from the RNA-SEQ on White Adipose Tissue Between NormalMg-HFD and LowMg-HFD.
A negative $\log_2$ fold change indicates a higher expression in the NormalMg-HFD group compared to the LowMg-HFD.

**GO-Term; 20 lowest *p*-values**	Corrected *p*-value	Log_2 Fold change
Translational initiation	3.5E-06	-0.26
Protein transport	6.5E-06	-0.12
SRP-dependent cotranslational protein targeting to membrane	9.2E-06	-0.30
Intracellular protein transport	1.1E-05	-0.15
RRNA processing	1.4E-05	-0.21
Viral transcription	7.1E-05	-0.31
Cell division	7.7E-05	-0.15
Cytoplasmic translation	7.7E-05	-0.21
Nuclear-transcribed mRNA catabolic process, nonsense-mediated decay	7.7E-05	-0.24
Cell proliferation	8.2E-05	-0.17
Innate immune response	5.9E-04	-0.25
Inflammatory response	9.1E-04	-0.28
Antigen processing and presentation of exogenous peptide antigen via MHC class II	9.1E-04	-0.43
Adaptive immune response	1.9E-03	-0.45
Formation of translation preinitiation complex	2.8E-03	-0.15
Positive regulation of I-kappaB kinase/NF-kappaB signalling	3.7E-03	-0.19
Translation	5.7E-03	-0.11
Cell redox homeostasis	6.3E-03	-0.16
Fc-gamma receptor signalling pathway involved in phagocytosis	7.9E-03	-0.34
Phagolysosome assembly	7.9E-03	-0.76
**GO-Term; 20 highest fold change (decreased in NormalMg-HFD compared to LowMg-HFD)**	Corrected *p*-value	Log_2 Fold change
Positive regulation of cholesterol esterification	0.57	1.00
Positive regulation of heat generation	0.55	0.68
Negative regulation of neurotrophin TRK receptor signalling pathway	0.74	0.67
Positive regulation of double-strand break repair	0.74	0.67
L-glutamate import	0.62	0.65
Positive regulation of renal sodium excretion	0.65	0.63
Drug metabolic process	0.40	0.62
DNA dealkylation involved in DNA repair	0.76	0.61
Angiotensin mediated vasoconstriction involved in regulation of systemic arterial blood pressure	0.76	0.61
Desmosome assembly	0.36	0.61
Regulation of cysteine-type endopeptidase activity involved in apoptotic process	0.77	0.61
Amine metabolic process	0.71	0.60
Positive regulation of dopamine metabolic process	0.66	0.57
Adenylate cyclase-activating adrenergic receptor signalling pathway	0.73	0.57
Negative regulation of dopamine secretion	0.68	0.55
Regulation of cardiac conduction	0.59	0.55
Response to ozone	0.83	0.55
Leucine catabolic process	0.38	0.54

Angiotensin-activated signalling pathway	0.80	0.52
Positive regulation of heart rate	0.55	0.52
Regulation of fat cell differentiation	0.37	0.52
**GO-Term; 20 lowest fold change (increased in NormalMg-HFD compared to LowMg-HFD)**	Corrected *p*-value	Log_2 Fold change
Positive regulation of T cell differentiation in thymus	0.51	-1.11
Negative regulation of B cell receptor signalling pathway	0.21	-1.09
Positive regulation of type III hypersensitivity	0.05	-1.07
Cell activation	0.70	-1.06
Mast cell degranulation	0.49	-1.06
Antigen processing and presentation of exogenous peptide antigen via MHC class I	0.11	-1.02
Positive regulation of type I hypersensitivity	0.16	-1.02
Sphingosine metabolic process	0.67	-1.02
Positive regulation of CD4-positive, alpha-beta T cell differentiation	0.24	-0.99
Positive regulation of type IIa hypersensitivity	0.26	-0.95
Toll-like receptor 7 signalling pathway	0.32	-0.94
Negative regulation of cell proliferation involved in contact inhibition	0.48	-0.92
Positive regulation of neutrophil chemotaxis	0.11	-0.86
Negative regulation of trophoblast cell migration	0.78	-0.84
Cellular extravasation	0.49	-0.82
Relaxation of cardiac muscle	0.46	-0.82
Leukocyte migration involved in inflammatory response	0.37	-0.80
Branching involved in prostate gland morphogenesis	0.50	-0.80
Negative regulation of mast cell degranulation	0.37	-0.79
Negative regulation of T cell mediated cytotoxicity	0.62	-0.79
Activated T cell proliferation	0.27	-0.79
